# Long‐Term Safety and Clinical Effects of Nilotinib in Parkinson's Disease

**DOI:** 10.1002/mds.28389

**Published:** 2020-11-20

**Authors:** Fernando L. Pagan, Barbara Wilmarth, Yasar Torres‐Yaghi, Michaeline L. Hebron, Sanjana Mulki, Dalila Ferrante, Sara Matar, Jaeil Ahn, Charbel Moussa

**Affiliations:** ^1^ Translational Neurotherapeutics Program, Laboratory for Dementia and Parkinsonism, Department of Neurology Georgetown University Medical Center Washington District of Columbia USA; ^2^ Movement Disorders Clinic, Department of Neurology MedStar Georgetown University Hospital Washington District of Columbia USA; ^3^ Department of Biostatistics, Bioinformatics and Biomathematics Georgetown University Medical Center Washington District of Columbia USA

**Keywords:** Nilotinib, Parkinson's disease, UPDRS, PDQ‐39, MoCA

## Abstract

**Background:**

Nilotinib is US Food and Drug Administration–approved for leukemia, and this open‐label study investigated the safety, tolerability, and potential clinical effects of nilotinib in medically optimized patients with Parkinson's disease.

**Objectives:**

Safety and tolerability were the primary objectives, and clinical outcomes were exploratory.

**Methods:**

A total of 63 patients completed a 15‐month phase 2, double‐blind, placebo‐controlled study and were rerandomized 1:1 into an open‐label study of nilotinib 150 mg versus 300 mg for 12 months.

**Results:**

Nilotinib was safe and tolerated, and no adverse effects seemed to be related to the drug, and no differences in adverse events were observed between groups. Exploratory clinical outcomes showed that nilotinib 300 mg was remarkably stable from baseline to 27 months using partial and total Unified Parkinson's Disease Scale (UPDRS). Nilotinib 150 mg versus 300 mg, significantly declined using partial or the sum of UPDRS Parts I and II. There was no significant difference in nilotinib 150 mg versus 300 mg using UPDRS Part III (*on* levodopa) and total UPDRS Parts I to III. Subgroup analysis showed that late‐start nilotinib 150 mg significantly worsened using the sum of UPDRS Parts II + III and total UPDRS Parts I to III compared with late‐start nilotinib 300 mg. Quality of life using the Parkinson's Disease Questionnaire in nilotinib 150 mg significantly declined between 15 and 27 months compared with nilotinib 300 mg, and there was no change in cognition using the Montreal Cognitive Assessment between groups.

**Conclusions:**

This study provides evidence that nilotinib is safe and tolerated in Parkinson's disease. The exploratory clinical data will inform an adequately powered larger study to evaluate the efficacy of nilotinib 300 mg in Parkinson's disease. © 2020 The Authors. *Movement Disorders* published by Wiley Periodicals LLC on behalf of International Parkinson and Movement Disorder Society

Parkinson's disease (PD) is a neurodegenerative disorder with motor and nonmotor symptoms, loss of midbrain dopamine neurons, and accumulation of misfolded α‐synuclein. Discoidin domain receptors (DDRs) are receptor tyrosine kinases that are overexpressed in the midbrain of postmortem patients with PD.^1^ DDR knockdown with *shRNA* in vivo and in vitro^1^ and pharmacological inhibitors of DDRs, including nilotinib,^2^ increase dopamine levels and reduce α‐synuclein, hyper‐phosphorylated tau (p‐tau), and other neurotoxic proteins in several models of neurodegeneration.^2^, ^3^, ^4^, ^5^, ^6^, ^7^, ^8^, ^9^ Partial or complete deletion or inhibition of DDR1 in a mouse model challenged with α‐synuclein increases autophagy and reduces inflammation and neurotoxic proteins.^10^ Nilotinib (Tasigna, AMN107, Novartis) is a potent tyrosine kinase inhibitor of half‐maximal inhibitory concentration (IC_50_) of DDR1 at 1 nM.^2^, ^11^, ^12^, ^13^, ^14^, ^15^, ^16^ Nilotinib less preferentially inhibits the nonreceptor tyrosine kinase Abelson (IC_50_ > 20 nM)^11^, ^12^, ^13^, ^16^ and is US Food and Drug Administration (FDA)–approved for the treatment of Philadelphia chromosome positive chronic myeloid leukemia at oral doses of 300 mg twice daily.^11^, ^12^, ^13^ Our previous studies indicate that oral treatment with nilotinib, 150 or 300 mg, results in cerebrospinal fluid (CSF) maximum concentration of 2 to 4.7 nM, respectively, in patients with Alzheimer's disease (AD)^17^ and PD,^18^, ^19^, ^20^ therefore achieving a pharmacologically adequate concentration that would inhibit DDR1. Oral treatment with nilotinib increases central nervous system (CNS) dopamine and reduces p‐tau levels in a dose‐dependent manner.^17^, ^18^, ^19^, ^20^ Nilotinib 150 mg reduces oligomeric α‐synuclein.^19^ Nilotinib attenuates hippocampal atrophy and reduces CSF amyloid and plaque burden in AD,^17^ independent of Abelson inhibition.^17^, ^19^ These results are consistent with animal data showing that treatment with nilotinib reduces neurotoxic proteins via autophagy.^4^, ^6^, ^7^, ^9^

This is an open‐label extension (OLE) of a phase 2, double‐blind (DB), placebo‐controlled study that randomly assigned 75 participants 1:1:1 into placebo:150:300 mg nilotinib for 1‐year and 3‐month washout (15 months).^19^ At 15 months, participants were randomly reassigned to nilotinib 150 mg versus 300 mg for 12 months. Safety and tolerability were primary objectives. Exploratory outcomes included the potential effect of nilotinib on motor, cognitive, and functional decline in patients with moderately severe PD.

## 1 Subjects and Methods

### 1.1 Study Design

This is an OLE of a DB phase 2, placebo‐controlled study that randomly assigned 75 participants 1:1:1 into placebo:150:300 mg nilotinib for 1‐year and 3‐month washout (15 months).^19^ This study is part of a clinical trial that included (stage 1) an open‐label, random single dose (RSD) to perform a physiologically based population pharmacokinetics/pharmacodynamics study.^20^ Following RSD, the same participants were randomly assigned 1:1:1 into 3 groups (n = 25) and received placebo, 150 or 300 mg nilotinib (stage 2) once daily for 1 year followed by a 3‐month washout period (15 months).^19^ Participants who completed the DB study and still met the inclusion criteria provided written consent at 15 months (end of the washout) and were randomly reassigned to an OLE of nilotinib 150 mg versus 300 mg for 12 months (stage 3); hence the study duration is 27 months (Fig. S1).

### 1.2 Objectives

The primary objective of this study is to evaluate the safety and tolerability of nilotinib in individuals with PD. Safety was measured using the occurrence of adverse events (AEs) and serious adverse events (SAEs) deemed to be possibly, probably, or definitely related to the study drug. AEs were defined as QTc prolongation, myelosuppression, hepatotoxicity, and pancreatitis. Tolerability for a given participant was defined as the ability of participants to remain on treatment. Overall tolerability of the drug was defined as an acceptable number of up to 25% discontinuations. An exploratory objective also included clinical assessments of motor and nonmotor functions as measured by change from baseline to 27 months and 15 to 27 months on the Movement Disorders Society–Unified Parkinson's Disease Scale (MDS‐UPDRS), Montreal Cognitive Assessment (MoCA), and Parkinson's Disease Questionnaire ‐ 39 items (PDQ‐39).

### 1.3 Participants

All participants were optimized on PD medications, including levodopa and/or dopamine agonists approximately 1 to 2 months before consenting in writing and screening. Some patients (13.3%) had deep brain stimulation (DBS) prior to enrollment and were stable and met the inclusion criteria. At baseline, participants were confirmed to have PD according to the UK Brain Bank diagnostic criteria with Hoehn and Yahr (H&Y) stages 2.5 to 3, MDS‐UPDRS Part III motor score 20 to 40, and MoCA score ≥ 22 as we previously reported.^19^ Participants in the OLE study were stable on optimal PD medications, including levodopa, dopamine agonists (bromocriptine [Parlodel, US Pharms Holdings], pramipexole [Mirapex, Boehringer Ingelheim Pharmaceuticals, Inc.], ropinirole [Requip, GlaxoSmithKline], and rotigotine [Neupro, UCB]) and monoamine oxidase (MAO)‐B inhibitors (Azilect, which was not allowed in the DB study). Other medications such as acetylcholinesterase inhibitors (AChEI), including rivastagmine (Excelon Patch) were also used. Therapeutic doses of selective serotonin reuptake inhibitors, including citalopram (Celexa, Allergan Sales LLC; Cipramil, lundbeck), escitalopram (Lexapro, Forest Laboratories; Cipralex, Lundbeck Canada Inc.), fluoxetine (Prozac, Eli Lilly and Company), paroxetine (Paxil, SmithKline Beecham) and sertraline (Zoloft, Pfizer), and serotonin‐norepinephrine reuptake inhibitors, duloxetine (Cymbalta, Eli Lilly and Company), and venlafaxine (Effexor Wyeth Pharms Inc.) were allowed.

## 2 Randomization

This study employed a block randomization using *blockrand* function in R software (version 3.4) to randomly assign 63 participants into the 2 treatment groups. The block size varies between 6 to 12, and the randomization was done within blocks to ensure a balance in sample sizes across groups of blocks^21^; and the treatment proceeded in an open‐label manner so that site staff, investigators, raters, participants, and caregivers knew the dose. Medications were labeled by Georgetown University Medical Center (GUMC) Clinical Research Unit (CRU) with a package medical identification number (patient identification). Participants retained the same specific identification number (patient identification) that was assigned to them during the DB, placebo‐controlled treatment.

### 2.1 Standard Protocol Approvals and Registrations

This is a single‐center study that was conducted by the Translational Neurotherapeutics Program at GUMC. This study was conducted in accordance with Good Clinical Practice guidelines and was approved by the institutional review board (2016–0380) at GUMC. The study was conducted under US FDA Investigational New Drug 123183.

## 3 Clinical Assessments

All participants were tested in the *on* state <2 hours since the last dose of levodopa. A single rater conducted all clinical exams in all participants across all study visits, and *on* state was also verified with the participant and the objective report of study partner and study investigator/rater. Clinical assessments were performed at baseline and 15 and 27 months using the PDQ‐39, MoCA, and MDS‐UPDRS.

## 4 Measurement of Human MAO A and B

CSF was collected at baseline and 12 months during the DB phase 2 study as previously indicated.^19^, ^20^ Briefly, CSF was added to enzyme‐linked immunosorbent assay wells precoated with target MAOs and biotin‐tagged specific antibodies for human MAO‐A (LifeSpan Biosciences, Catalog No. LS‐F10846) or MAO‐B (My BioSource, Catalog No. MBS70029). avidin–horseradish peroxidase (HRP) was added to capture biotin tagged detection antibody and incubated. A substrate solution of 3,3′,5,5′‐Tetramethylbenzidine was added to conjugate avidin–HRP, leading to color development that was read at 450 nm using a microplate reader according to manufacturers' protocol.

## 5 Data Analysis and Statistical Plan

Baseline descriptive statistics such as mean ± SD for continuous demographic and dose variables and frequencies of safety endpoints were descriptively summarized for the 150 and 300 mg OLE groups. The frequencies and proportions of SAEs and nonserious AEs among the 2 groups were tabulated and compared using the Fisher exact test.

The 2 OLE study subgroups were further classified into the following 6 groups depending on treatment types during the DB phase: group A includes patients who received placebo in DB and 150 mg nilotinib in OLE (late‐start low dose); group B includes patients who received placebo in DB and 300 mg nilotinib in OLE (late‐start high dose); group C includes nilotinib 150 mg in both DB and OLE (early‐start low dose); group D includes nilotinib 300 mg in DB and OLE (early‐start high dose); group E includes patients who increased the dose from 150 mg nilotinib in DB to 300 mg nilotinib in OLE (dose increase); group F includes patients who received 300 mg nilotinib in DB and 150 mg nilotinib in OLE (dose reduction).

Exploratory clinical endpoints by the 6 groups at baseline, 15 months, and 27 months were summarized using sample mean ± SD. Within each group, changes in clinical endpoints between baseline and 15 months, baseline and 27 months, and 15 and 27 months were tested using a paired Wilcoxon rank‐sum test and their Wilcoxon 95% confidence intervals, respectively. Using the Wilcoxon test, the within‐group changes in each clinical endpoint between baseline and 15 months, baseline and 27 months, and 15 and 27 months were compared between groups A and B, groups A and C, and groups B and D, respectively. Considering that clinical endpoint comparisons are exploratory and hypothesis generating, a 2‐sided type I error of 5% was used without no multiple testing corrections. All statistical analyses were performed using R version 3.40.

### 5.1 Data Sharing

The final data, study protocol and all interpretations will be fully available to the scientific and nonscientific community and clinicians. Investigators adhered to the Privacy Rule under the Health Insurance Portability and Accountability Act.

## 6 Results

### 6.1 Patients, Demographics, Enrollment, and Randomization

**FIG. 1:**
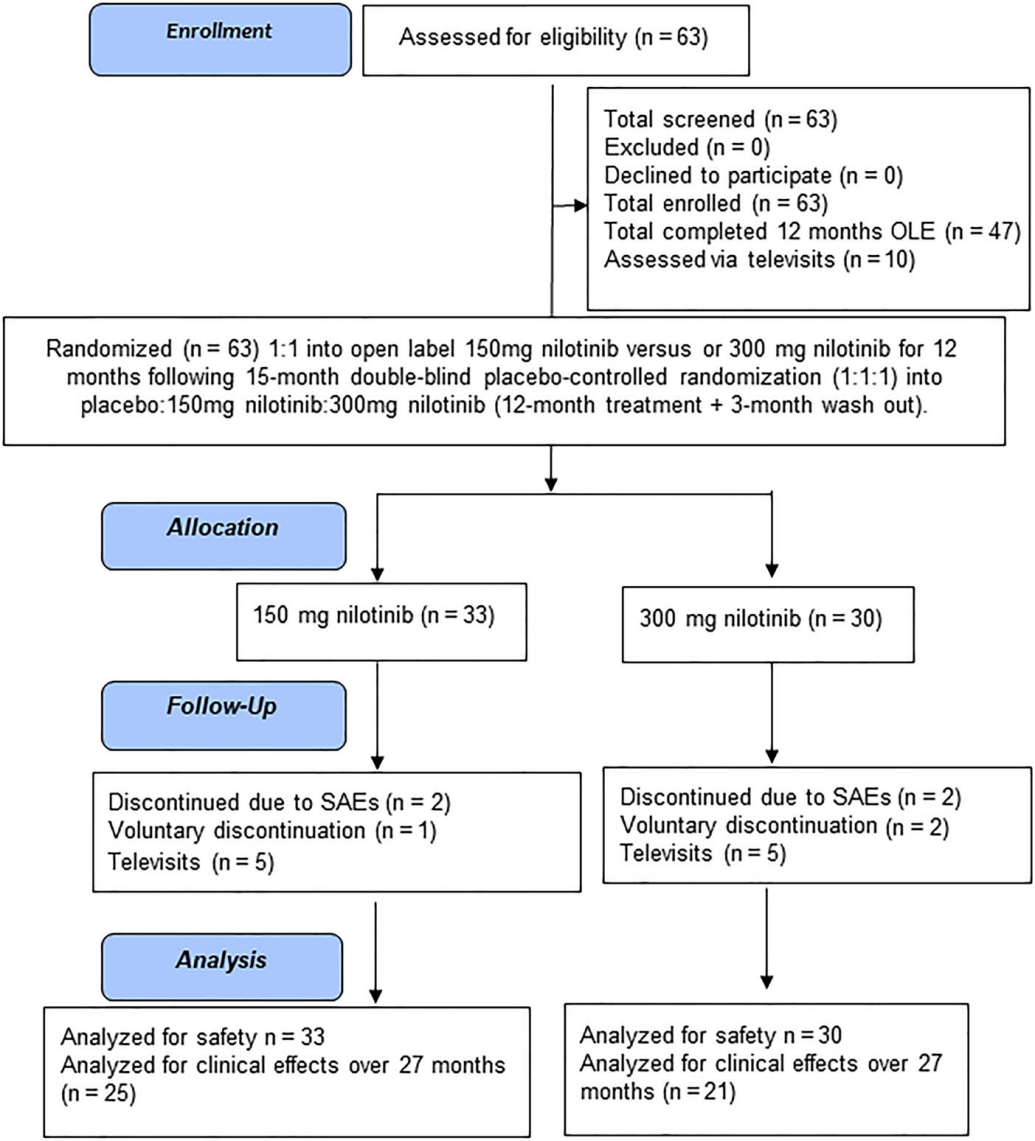
Consolidated Standards of Reporting Trials of randomized open‐label extension of 150 and 300 mg nilotinib in moderately advanced Parkinson's disease. SAEs, serious adverse events. [Color figure can be viewed at wileyonlinelibrary.com]

A total of 63 participants completed the DB, placebo‐controlled period and enrolled at the 15‐month washout visit (Fig. 1 and Table 1) in the OLE study. Participants were 70.12 ± 7.8 (mean ± SD) years of age and included 16 women and 47 men and 90.5% of participants completed the OLE. There were no dropouts attributed to lack of drug tolerability. All participants were H&Y stages 2.5 to 3 with a disease duration of 12.12 ± 5.67 (mean ± SD) years nilotinib 150 mg, and 10.48 ± 4.21 years (mean ± SD) years in nilotinib, 300 mg in. Of the participants, 3 dropped out of the 150 mg group, and 2 were attributed to SAEs, including 1 esophageal carcinoma and 1 cervical cord compression. One participant withdrew voluntarily (Fig. 1). A total of 4 participants dropped out of the 300 mg group; 2 were attributed to SAEs, including 1 renal failure and another diagnosed with non–ST‐segment elevation myocardial infarction (NSTEMI). One participant self‐withdrew from the study voluntarily, and another withdrew because of concerns about travel during coronovarirus disease (COVID)–19. A total of 10 participants, 5 in each group, were unable to come to the CRU, which closed down because of COVID‐19, and were assessed via televisits and closed‐out between March and July 2020.

**TABLE 1:** Demographics and enrollment summary

Demographics	Nilotinib 150 mg	Nilotinib 300 mg
Total enrolled	N = 33	N = 30
Number of dropouts	3	4
Data loss because of televisits at or before 12 months OLE (because of COVID‐19)	5	5
Total analyzed for clinical effects	25	21
Average age (y) ± SD	71.41 ± 6.49	68.83 ± 9.06
Hoehn and Yahr stage	2.5–3	2.5–3
BMI ± SD	26.59 ± 4.4	26.1 ± 4.43
Male/female	23/10	24/6
Race	32 white/1 Asian	29 white/1 Asian
Duration of disease (y), mean ± SD	12.12 ± 5.67	10.48 ± 4.21
Hoehn and Yahr stage	2.5–3	2.5–3

Abbreviations: OLE, open‐label extension; COVID‐19, coronovirus disease 2019; BMI, body mass index.

### 6.1 Nonserious AEs

The total number of AEs was 74 with nilotinib 150 mg and 58 AEs with nilotinib 300 mg (Table 2), but there was no significant (*P* = 0.42) difference between the groups. Falls were common AEs with nilotinib 150 mg (45.4%) and 300 mg (43%), but there was no significant difference (*P* = 0.86) between the groups. Other common AEs were skin lesions (24%–30%) attributed to local infections or healing wounds and upper respiratory tract infections in nilotinib 150 mg (12% and 24%, respectively) and nilotinib 300 mg (30% and 26.7%, respectively), but there was no significant difference (*P* = 0.6 and *P* = 0.14, respectively) between the groups. Less common AEs were pain and surgery (15%) and urinary tract infection (UTI) with nilotinib 150 mg (12%). All other AEs were ≤ 10% in both groups. There was no QTc prolongation across all visits in both groups (Tables S1 and S2).

**TABLE 2:** Summary of all nonserious AEs and SAEs reported according to preferred terms in all treatment groups

All AEs	All AEs
150 mg nilotinib, no. of events (%)	300 mg nilotinib, no. of events (%)
Stent 1 (3)	Atrial flutter 1 (3.3)
Hypertension 1 (3)	Stent placement 1 (3.3) RBBB 1 (3.3)
Cataract 2 (6)	Cataract 1 (3.3)
Eye laceration 1 (3)	Conjunctivitis 1 (3.3)
Diarrhea 1 (3) Acid reflux 2 (6) Nausea 1 (3) Abdominal pain 3 (9)	Acid reflux 1 (3.3)
Pain 5 (15) Surgery 5 (15) Edema 1 (4.2)	Pain 2 (6.7) Surgery 2 (6.7) Torn ligament 2 (3.3) Tendonitis 1 (3.3) Bursitis 2 (6.7)
Sciatica 1 (3) REM sleep 1 (4.2) DBS battery replacement 1 (3)	Sciatica 1 (3.3)
Hallucinations 1 (3) Cognitive decline 1 (3)	
UTI 4 (12) Prostate 1 (3)	UTI 1 (3.3) Prostate 1 (3.3)
URI 4 (12) Pneumonia 2 (6)	UR1 8 (26.7) Pneumonia 2 (6.7)
Lesion 8 (24) Lumps 2 (6) Itchy skin 1 (3)	Lesion 9 (30) Lumps 3 (10) Carcinoma excision 2 (6.7) Redness 1 (3.3)
Falls 15 (45.4) Dehydration 1 (3) Infection 3 (9) Weakness 1 (3) Flu‐like symptoms 1 (3) Fever 1 (3)	Falls 13 (43) Infection 1 (3.3) Food poisoning 1 (3.3)
All SAEs	
Orthostatic hypotension 1 (3)	NSTEMI 1 (3.3)
Catheterization 1 (3)	Stenting/pacemaker 1 (3.3)
Hallucinations 1 (3)	
UTI 1 (3)	Renal failure 1 (3.3)
pneumonia 1 (3)	Dysphagia 1 (3.3)
Fever 1 (3)	
DBS surgery site infection 1 (3) C4 cord compression 1 (3)	
Esophageal carcinoma 1 (3)	

Abbreviations: AEs, adverse events; SAEs, serious adverse events; RBBB, right bundle branch block; REM, rapid eye movement; DBS, deep brain stimulation; UTI, urinary tract infection; URI, upper respiratory tract infection NSTEMI, non–ST‐segment elevation myocardial infarction.

### 6.2 Serious Adverse Events

The total number of SAEs was 9 with nilotinib 150 mg and 4 with nilotinib 300 mg (Table 2), but there was no significant difference (*P* = 0.22) between the groups. In the nilotinib 150 mg group, 1 participant was hospitalized for orthostatic hypotension and another had a cardiac catheterization procedure, but they both continued the treatment and completed the study. Two other SAEs resulted in patients' withdrawal, 1 attributed to cervical cord compression and subsequent withdrawal by the principal investigator (PI) and another because of esophageal carcinoma. Other patients were also hospitalized for hallucinations, UTIs, pneumonia, fever, and DBS surgery site infection. In the nilotinib 300 mg group, 1 patient withdrew from the study because of an NSTEMI and another was withdrawn by the PI as a result of renal failure. One had a pacemaker placed and continued the study, and another was hospitalized because of dysphagia.

### 6.3 Clinical Outcomes

#### 6.3.1 *MDS‐UPDRS in Nilotinib Groups*

**FIG. 2:**
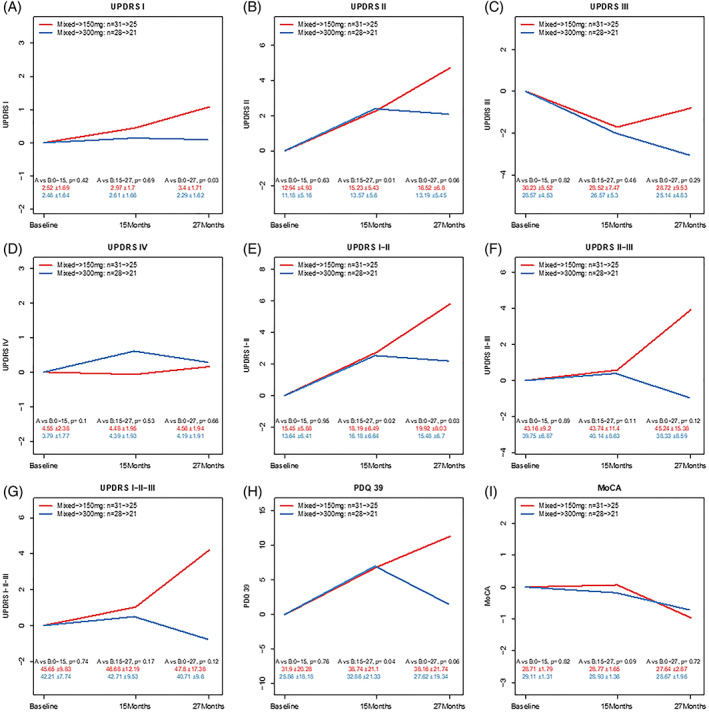
Graphs represent Movement Disorders Society Unified Parkinson's Disease Rating Scale (UPDRS) (**A**) Part I (cognition and mentation), (**B**) Part II (activities of daily living), (**C**) Part III (motor symptoms), (**D**) Part IV, (**E**) sum of Parts I + II, (**F**) sum of Parts II + III, and (**G**) total Parts I to III; (**H**) Parkinson's Disease Questionnaire (PDQ)‐39; and (**I**) Montreal Cognitive Assessment (MoCA). Mixed: participants received either placebo, 150 mg, or 300 mg nilotinib in the double‐blinded phase (15 M) versus drug in open‐label extension (12 m). [Color figure can be viewed at wileyonlinelibrary.com]

Assessment of cognition and mentation using UPDRS Part I shows that nilotinib 300 mg is stable between baseline and 27 months (Fig. 2A, Table S3), but there was a significant decline (*P* = 0.03) in nilotinib 150 mg versus 300 mg (Fig. 2A, Table S3). Assessment of activities of daily living using UPDRS Part II (Fig. 2B, Table S3) indicates that nilotinib 300 mg was stable between baseline and 27 months. However, at 15 to 27 months, there was a significant decline (*P* = 0.01) in UPDRS II in nilotinib 150 mg versus 300 mg (Table S3). Assessment of motor symptoms (*on* levodopa) using UPDRS Part III (Fig. 2C, Table S3) shows no significant differences at baseline to 27 months in nilotinib 150 mg versus 300 mg, indicating motor stability. There was no difference in UPDRS Part IV (Fig. 2D, Table S3) between nilotinib 150 mg versus 300 mg. There was a significant decline in the sum of UPDRS Parts I + II (Fig. 2E, Table S3) in nilotinib 150 mg versus 300 mg between baseline and 27 months (*P* = 0.03) and also 15 to 27 months (*P* = 0.02). No significant changes were seen in the sum of UPDRS Parts II + III (Fig. 2F, Table S3) and total UPDRS Parts I to III (Fig. 2G, Table S3).

**FIG. 3:**
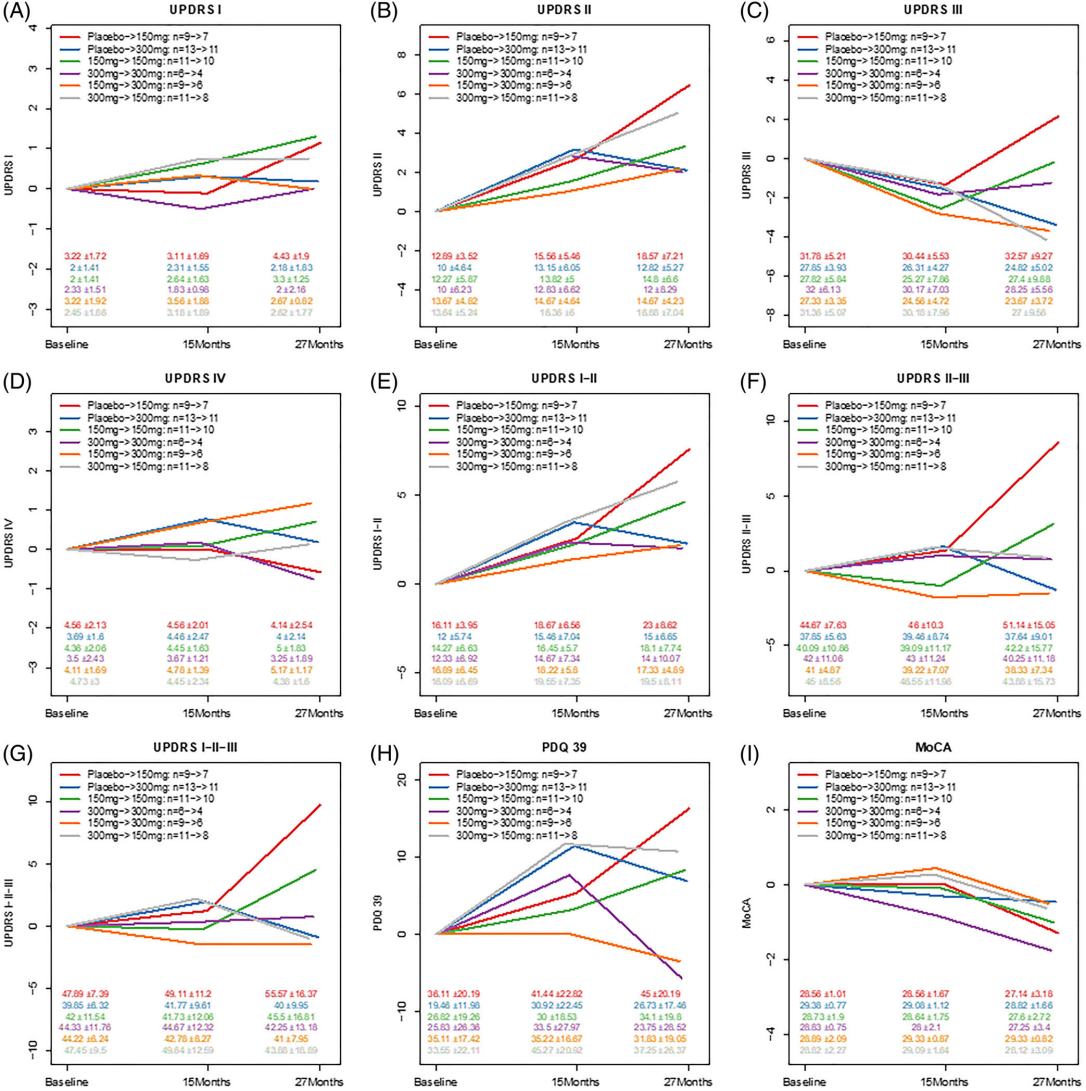
Graphs represent Movement Disorders Society Unified Parkinson's Disease Rating Scale (UPDRS) (**A**) Part I (cognition and mentation), (**B**) Part II (activities of daily living), (**C**) Part III (motor symptoms), (**D**) Part IV, (**E**) sum of Parts I + II, (**F**) sum of Parts II + III, and (**G**) total Parts I to III; (**H**) Parkinson's Disease Questionnaire (PDQ)‐39; and (**I**) Montreal Cognitive Assessment (MoCA). Groups A and B received placebo in double‐blind phase and 150 or 300 mg in open‐label extension (OLE), respectively (late start). Group C received 150 mg in double‐blind phase and 150 mg in OLE (early start). Group D received 300 mg in double‐blind phase and 300 mg in OLE (early start). Group E received 150 mg in double‐blind phase and 300 mg in OLE. Group F received 300 mg in the double‐blind phase and 150 mg in OLE. [Color figure can be viewed at wileyonlinelibrary.com]

Subgroup analysis of late‐start nilotinib 150 mg (group A) and 300 mg (group B) versus early‐start nilotinib 150 mg (group C) and 300 mg (group D) showed that nilotinib 300 mg was remarkably stable (Fig. 3, Tables S4 and S5). There was no difference in UPDRS Part I between all groups (Fig. 3A, Table S5). At 15 to 27 months, late‐start nilotinib 150 mg significantly declined (*P* = 0.02) compared with late‐start nilotinib 300 mg using UPDRS Part II (Fig. 3B, Table S5), but there were no differences in UPDRS Part III (Fig. 3C) and UPDRS Part IV (Fig. 3D). At 15 to 27 months, the sum of UPDRS Parts I + II (Fig. 3E, Table S5) significantly declined (*P* = 0.01) in nilotinib 150 mg versus 300 mg. At 15 to 27 months, the sum of UPDRS Parts II + III (Fig. 3F, Table S5) and total UPDRS Parts I to III (Fig. 3G, Table S5) also significantly declined (*P* = 0.01 and *P* = 0.01, respectively) in nilotinib 150 mg versus 300 mg.

#### 6.3.2 *PDQ‐39 and MoCA*

Assessment of quality of life (QoL) using the PDQ‐39 (Fig. 2H, Table S3) indicates a significant difference (*P* = 0.04) at 15 to 27 months with nilotinib 150 mg versus 300 mg. Late‐start nilotinib 150 mg significantly declined (*P* = 0.03) compared with late‐start nilotinib 300 mg using the PDQ‐39 at 15 to 27 months (Fig. 3H, Table S5). Cognitive evaluation using MoCA (Fig. 2I, Table S3) showed that nilotinib 150 mg or 300 mg were clinically stable at baseline to 27 months, and there were no differences in MoCA (Fig. 3I) between the early‐start and late‐start groups.

### 6.5 Levodopa Equivalent Daily Dose

Levodopa equivalent daily dose (LEDD) indicates whether patients changed their PD medications between baseline and 15 and 27 months (Table S6). A total of 6 patients (24%) had DBS in the nilotinib 150 mg group versus 5 patients (24%) in the 300 mg group (*P* = 0.54). Although LEDD was increased with nilotinib 150 mg (26%) and 300 mg (30%) between baseline and 27 months, there was no significant difference (*P* = 0.68) in LEDD increase between the groups. There was also an increase in LEDD in both 150 mg (22%) and 300 mg (19%) in the late‐start nilotinib groups, but there was no significant difference between the groups (*P* = 0.89). There was a significantly higher level (*P* = 0.01) of AChEI in nilotinib 150 mg (24%) versus 300 mg (6%) between baseline and 27 months.

### 6.6 MAO Activity

Oral treatment with nilotinib increases CNS dopamine in a dose‐dependent manner.^17^, ^18^, ^19^, ^20^, ^22^ We measured the activity of MAOs that catabolize dopamine to better understand the reason of the change in CSF dopamine metabolites.^17^, ^18^, ^19^, ^20^, ^22^ MAO‐A activity between baseline and 12 months in the DB, placebo‐controlled study was reduced (albeit nonsignificant) in nilotinib 150 mg (−240%, *P* = 0.31) and 300 mg (−379%, *P* = 0.21) versus placebo (Fig. S2A). There was no difference in MOA‐B from baseline to 12 months (Fig. S2B) between the groups.

## 7 Discussion

This OLE of a phase 2 study to evaluate nilotinib safety in patients with PD shows that nilotinib is tolerated and safe. We previously showed that 88% of patients completed the DB, placebo‐controlled 15‐month period,^19^ and 90.5% completed the OLE with no dropouts attributed to intolerability. There were 2 dropouts in the nilotinib 150 mg group as a result of SAEs, including 1 patient who was diagnosed with esophageal carcinoma and withdrew from the study and another had cervical cord compression that resulted in multiple falls and withdrawal by the PI. In the nilotinib 150 mg group, 1 participant voluntarily withdrew from the study. There were 2 SAEs in the nilotinib 300 mg group, including 1 patient who had preexisting renal insufficiency and was withdrawn (by the PI) from the study as a result of renal failure. Another patient received placebo in the DB period and was diagnosed with NSTEMI and self‐discontinued from the study in the first week (fourth day) of OLE. There were more AEs and SAEs, although nonsignificant, with nilotinib 150 mg compared with 300 mg, indicating that the observed SAEs are not related to increased drug dose and were probably the result of preexisting or other conditions. As demonstrated in the DB, placebo‐controlled study,^19^ and also in a phase 2 nilotinib study in AD,^17^ there was no QTc prolongation in any patient. In the DB period, there was a significant increase in the total number of SAEs in the nilotinib groups, but no significant difference was seen in cardiovascular SAEs, number of falls (albeit increased in the DB nilotinib groups), or the total number of AEs between all the groups.^19^ This OLE study demonstrates less SAEs in nilotinib 300 mg versus 150 mg and shows that long‐term nilotinib treatment does not result in increased number of falls with either treatment—compared with the number of falls in the placebo group during the DB period—or dose. Furthermore, 1‐year nilotinib treatment of patients with AD did not result in any SAE.^17^ Collectively, these findings suggest that nilotinib is safe and that the AEs observed in these studies were either attributed to the nature of the disease, commonly occur in the elderly population, or were unrelated to nilotinib treatment.

This is a phase 2 study that is underpowered (by design) to detect nilotinib effects on clinical outcomes, but the adaptive design of this OLE allows the assessment of exploratory measures of motor and nonmotor symptoms to guide the evaluation of nilotinib safety and efficacy in larger phase 3 studies. Exploratory clinical measures show that nilotinib 300 mg results in remarkably stable UPDRS scores, including cognition (Part I), activities of daily living (Part II), and motor (Part III) symptoms over 27 months. This stability in UPDRS scores, particularly in Parts I and II, was not observed with nilotinib 150 mg. The effects of nilotinib 300 mg compared with 150 mg on activities of daily living using the UPDRS Part II were echoed in health‐related QoL using the PDQ‐39, which showed a clinically meaningful change^23^ of 10 to 20 points between the groups. Furthermore, there was no cognitive change in nilotinib 150 and 300 mg using MoCA scores, which averaged 28.59/30 at the end of 27 months, indicating stable cognition in both nilotinib groups. These exploratory clinical data support the evaluation of the efficacy of nilotinib in more adequately powered studies.

Optimization of PD treatment, including 13.3% DBS and the small sample size in the DB, placebo‐controlled, study could affect UDPRS scores. In the OLE, 25% of nilotinib 150 and 300 mg groups were on DBS, and both groups equally increased LEDD (22%–27.8%) for 27 months. In the DB 15‐month period, no clinical worsening in UPDRS was observed in the nilotinib groups compared with placebo, which slightly worsened 2.47 points using total UPDRS Parts I to III^19^; but although nilotinib 300 mg maintained stability for 27 months, the nilotinib 150 mg group significantly worsened up to 10 points. Oral treatment with nilotinib increases CNS dopamine in a dose‐dependent manner in both patients with PD who receive levodopa as well as patients with AD who are levodopa naïve.^17^, ^18^, ^19^, ^20^ These results are consistent with our data showing a trend of dose‐dependent inhibition of MAO‐A in patients with PD, in agreement with previous data that nilotinib reduces dopamine catabolism in mice^4^, ^6^ and human.^17^, ^18^, ^19^, ^20^ Taken together, these findings suggest that nilotinib treatment in patients with early PD who are levodopa naïve should be evaluated to determine whether nilotinib stabilizes PD symptoms and/or eliminates or delays resumption of levodopa treatment.

Nilotinib may have potential long‐term effects on clinical outcomes as a result of disease modification in neurodegeneration. Nilotinib 150 mg significantly reduces oligomeric α‐synuclein in PD.^19^ Accumulation of oligomeric α‐synuclein results in age‐dependent impairment of dopamine release, but reduction of oligomeric α‐synuclein increases dopamine turnover.^24^ In patients with PD and Lewy body dementia, nilotinib affects CSF miRNAs that control autophagy and cellular transport genes, including synaptosome associated protein 25 (SNAP25) and vacuolar protein sorting (VPS).^10^ SNAP25 and VPS mediate the transport and fusion of the endosome, lysosome, and neurotransmitter vesicles with the plasma membrane,^10^, ^25^, ^26^, ^27^ thereby regulating autophagy and neurotransmitter release. These data are consistent with the increased level of dopamine metabolism in animals^7^, ^12^ and patients with PD^18^, ^19^, ^20^ and AD^17^ treated with nilotinib.^4^, ^6^, ^17^, ^18^, ^19^, ^20^, ^28^ Furthermore, nilotinib reduces the level of p‐tau in the CSF of patients with PD^19^ and AD^17^ and the brain of animal models of neurodegeneration.^3^, ^4^, ^5^, ^6^, ^19^, ^29^ Reduction of p‐tau in nilotinib‐treated animals enhances astrocyte activity and improves neurotransmitter balance.^7^ Autophagy clearance of α‐synuclein and p‐tau and regulation of SNAP25 and VPS are concurrent with improved astrocytic activity and balance of neurotransmitters.^4^, ^5^, ^7^, ^8^, ^30^ Nilotinib also attenuates hippocampal atrophy and significantly reduces CSF amyloid and brain plaque (via amyloid positron emission tomography) burden in patients with AD.^17^ Clinically, patients with AD who receive nilotinib 300 mg, but not 150 mg, exhibit behavioral mood swings, including agitation and irritability, which is consistent with the dose‐dependent increase of brain dopamine.^17^ These findings are in agreement with animal data showing that treatment with nilotinib reduces neurotoxic proteins via autophagy,^4^, ^6^, ^7^, ^9^ and these effects may lead to improvement of clinical outcomes, hence a potential long‐term disease modification. Collectively, nilotinib effects on neurodegenerative pathology, including PD, may be mediated by multiple changes, representing a “biomarker mix” that includes reduction of CSF oligomeric α‐synuclein and p‐tau, which affect the brain motor and nonmotor systems, and improvement of dopamine metabolism.^17^, ^18^, ^19^, ^20^, ^22^ These changes constitute an experimental biomarker system similar to the A/T/N system in AD^31^ that should be investigated in correlation with potential clinical outcomes to evaluate the disease‐modifying effects of nilotinib.

## 8 Conclusions

This OLE of a DB phase 2 study met its primary objective and showed that long‐term nilotinib treatment is safe and tolerated in patients with PD. Exploratory clinical outcomes support the evaluation of nilotinib 300 mg in a larger multicenter phase 3 study to determine its safety and efficacy in PD. This study is underpowered and was performed in a single center. The small number of patients in each treatment group prevents any meaningful analysis for multiple comparisons.

## Author Roles

(1) Research Project: A. Conception, B. Organization, C. Execution; (2) Statistical Analysis: A. Design, B. Review and Critique; (3) Manuscript: A. Writing of the First Draft. B. Review and Critique.

F.L.P.: 1C, 3B

B.W.: 1C, 3B

Y.T.‐Y.: 1C, 3B

M.L.H.: 1C, 3B

S. Mulki: 1C, 3B

D.F.: 3B

S. Matar: 1C, 3B

J.A.: 2A, 2B, 3B

C.M.: 1A, 1B, 3A, 3B

Full financial disclosures for the previous 12 months:

## Supporting information


**Figure S1.** A schematic representation of the 27‐month nilotinib study in patients with moderately severe Parkinson's. The study included a single random dose (RSD) of 5 groups who were then randomized into double‐blind, placebo‐controlled treatment for 1 year followed by 3 months follow‐up. At 15 months, participants were reconsented and rerandomized 1:1 to open‐label nilotinib 150 and 300 mg for 1 year to evaluate the long‐term effects (27 months) of niloitnib on safety. An exploratory objective was to determine potential long‐term clinical effects in mixed patients who received either nilotinib or placebo in the double‐blind study (mixed or early start) and late‐start nilotinib who received only placebo in the double‐blind treatment period.
**Figure S2.** Graph represents mean difference between Baseline and 12 months treatment of (A) monoamine oxidase (MAO)‐A and (B) MAO‐B activity in the cerebrospinal fluid of patients with Parkinson's treated with placebo (n = 21), 150 mg nilotinib (n = 21), or 300 mg nilotinib (n = 20).

**Table S1.** Summary of electrocardiogram values for all participants throughout all study visits showing no QTc prolongation in the 150 mg nilotinib group. The baseline QTc range for inclusion into this study was 350–460 milliseconds. A serious adverse event was defined as QTc prolongation ≥60 milliseconds from baseline of individual participants and to a value ≥480 milliseconds or QTcF prolongs ≥500 milliseconds. m, month; NC, noncompliance
**Table S2.** Summary of electrocardiogram values for all participants throughout all study visits showing no QTc prolongation in the 300 mg nilotinib group. The baseline QTc range for inclusion into this study was 350–460 milliseconds. A serious adverse event was defined as QTc prolongation ≥60 milliseconds from baseline of individual participants AND to a value ≥480 milliseconds or QTcF prolongs ≥500 milliseconds. m, month; NC, noncompliance.
**Table S3.** Pairwise comparison of changes in clinical endpoints between 150 mg open‐label extension group (mixed to 150 mg) and 300 mg open‐label extension group (mixed to 300 mg) across 2 visits; 15 months‐27 months, baseline‐15 months, baseline‐27 months.
**Table S4.** Mean changes in clinical endpoints within each group across 2 visits, 15 months and 27 months, between baseline and 15 months, and baseline and 27 months. Comparison were performed using Wilcoxon rank‐sum test and their 95% confidence intervals. Group A represents placebo to 150 mg (n = 9), B represents placebo to 300 mg (n = 13), C represents 150 mg to 150 mg (n = 11), D represents 300 mg to 300 mg (n = 6), E represents 150 mg to 300 mg (n = 9), F represents 300 mg to 150 mg (n = 11).
**Table S5.** Pairwise comparison of changes in clinical endpoints between groups A (placebo to 150 mg) and B (placebo to 300 mg), groups A and C (150 mg to 150 mg), and groups B and D (300 mg to 300 mg), across 2 visits; 15 months ‐27 months, baseline ‐15 months, baseline‐27 months.
**Table S6.** Levodopa Equivalent Daily Dose (LEDD) + Deep Brain Stimulation (DBS) + Acetylcholinesterase inhibitors (AChEI) in each group: (A) Placebo to 150 mg, (B) placebo to 300 mg, (C) mixed to 150 mg, (D) mixed to 300 mg nilotinib. Mixed = participants received either placebo, 150 mg, or 300 mg nilotinib in the double‐blinded phase (15 M) versus drug in open‐label extension (12 m). Placebo = participants received only placebo in the double‐blinded phase (15 M) versus drug in open‐label extension (12 M).
