# Supplementary material for: Long‐Term Safety and Clinical Effects of Nilotinib in Parkinson's Disease
Source: Mov Disord. 2020 Nov 20;36(3):740–9. doi: 10.1002/mds.28389 (PMC8048914; doi:10.1002/mds.28389)
Supplement: Supplementary file 1 — Figure S1. A schematic representation of the 27‐month nilotinib study in patients with moderately severe Parkinson's. The study included a single random dose (RSD) of 5 groups who were then randomized into double‐blind, placebo‐controlled treatment for 1 year followed by 3 months follow‐up. At 15 months, participants were reconsented and rerandomized 1:1 to open‐label nilotinib 150 and 300 mg for 1 year to evaluate the long‐term effects (27 months) of niloitnib on safety. An exploratory objective was to determine potential long‐term clinical effects in mixed patients who received either nilotinib or placebo in the double‐blind study (mixed or early start) and late‐start nilotinib who received only placebo in the double‐blind treatment period. Figure S2. Graph represents mean difference between Baseline and 12 months treatment of (A) monoamine oxidase (MAO)‐A and (B) MAO‐B activity in the cerebrospinal fluid of patients with Parkinson's treated with placebo (n = 21), 150 mg nilotinib (n = 21), or 300 mg nilotinib (n = 20). [file MDS-36-740-s002.docx]

**Suppl. Figure 1-** A schematic representation of the 27-month nilotinib study in moderately severe Parkinson’s patients. The study included a single random dose (RSD) of 5 groups, who were then randomized into double-blind, placebo-controlled treatment for 1 year followed by 3 months follow up. At 15 months participants were re-consented and re-randomized 1:1 to open label nilotinib 150mg and 300mg for one year to evaluate the long-term effects (27 months) of niloitnib on safety. An exploratory objective was to determine potential long-term clinical effects in mixed patients who received either nilotinib or placebo in the double-blind study (mixed or early start) and late-start nilotinib who received only placebo in the double-blind treatment period.

**Nilotinib, 150mg**

**Nilotinib, 200mg**

**Nilotinib, 300mg**

**Nilotinib, 400mg**

**Placebo**

**Nilotinib, 150mg**

**Nilotinib, 300mg**

**Nilotinib, 150mg**

**Nilotinib, 300mg**

**Placebo**

**Open Label Extension (12 months)**

**Primary Outcomes: Safety**

**Exploratory Outcomes:**

**Early start (Mixed to nilotinib)**

**Vs**

**Late start (Placebo to nilotinib)**

**Open Label Extension (12 months)**

**Primary Outcomes: Safety**

**Exploratory Outcomes:**

**Early start (Mixed to nilotinib)**

**Vs**

**Late start (Placebo to nilotinib)**

**Double Blind Outcomes**

**Primary: Safety, Tolerability and PK**

**Secondary: Biomarkers including,**

**dopamine metabolism (Go-NoGo)**

**Total and oligomeric alpha-synuclein and**

**Ttau/ptau**

**Exploratory:**

**MDS-UPDRS, PDQ39 and MoCA**

**RSD Outcomes**

**Population Based**

**PK/PD single dose**

**Double Blind Outcomes**

**Primary: Safety, Tolerability and PK**

**Secondary: Biomarkers including,**

**dopamine metabolism (Go-NoGo)**

**Total and oligomeric alpha-synuclein and**

**Ttau/ptau**

**Exploratory:**

**MDS-UPDRS, PDQ39 and MoCA**

**RSD Outcomes**

**Population Based**

**PK/PD single dose**

**Open Label Extension (12 months)**

**Primary Outcomes: Safety**

**Exploratory Outcomes:**

**Early start (Mixed to nilotinib)**

**Vs**

**Late start (Placebo to nilotinib)**

**Nilotinib, 150mg**

**Nilotinib, 300mg**

**Suppl. Figure 2-** Graph represents mean difference between Baseline and 12 months treatment of A) monoamine oxidase (MAO)-A and B) MAO-B activity in the CSF of Parkinson’s patients treated with placebo (n=21), 150mg nilotinib (n=21) and 300 mg nilotinib (n=20).

A

B
