# Supplementary material for: Long‐Term Safety and Clinical Effects of Nilotinib in Parkinson's Disease
Source: Mov Disord. 2020 Nov 20;36(3):740–9. doi: 10.1002/mds.28389 (PMC8048914; doi:10.1002/mds.28389)
Supplement: Supplementary file 2 — Table S1. Summary of electrocardiogram values for all participants throughout all study visits showing no QTc prolongation in the 150 mg nilotinib group. The baseline QTc range for inclusion into this study was 350–460 milliseconds. A serious adverse event was defined as QTc prolongation ≥60 milliseconds from baseline of individual participants and to a value ≥480 milliseconds or QTcF prolongs ≥500 milliseconds. m, month; NC, noncompliance Table S2. Summary of electrocardiogram values for all participants throughout all study visits showing no QTc prolongation in the 300 mg nilotinib group. The baseline QTc range for inclusion into this study was 350–460 milliseconds. A serious adverse event was defined as QTc prolongation ≥60 milliseconds from baseline of individual participants AND to a value ≥480 milliseconds or QTcF prolongs ≥500 milliseconds. m, month; NC, noncompliance. Table S3. Pairwise comparison of changes in clinical endpoints between 150 mg open‐label extension group (mixed to 150 mg) and 300 mg open‐label extension group (mixed to 300 mg) across 2 visits; 15 months‐27 months, baseline‐15 months, baseline‐27 months. Table S4. Mean changes in clinical endpoints within each group across 2 visits, 15 months and 27 months, between baseline and 15 months, and baseline and 27 months. Comparison were performed using Wilcoxon rank‐sum test and their 95% confidence intervals. Group A represents placebo to 150 mg (n = 9), B represents placebo to 300 mg (n = 13), C represents 150 mg to 150 mg (n = 11), D represents 300 mg to 300 mg (n = 6), E represents 150 mg to 300 mg (n = 9), F represents 300 mg to 150 mg (n = 11). Table S5. Pairwise comparison of changes in clinical endpoints between groups A (placebo to 150 mg) and B (placebo to 300 mg), groups A and C (150 mg to 150 mg), and groups B and D (300 mg to 300 mg), across 2 visits; 15 months ‐27 months, baseline ‐15 months, baseline‐27 months. Table S6. Levodopa Equivalent Daily Dose (LEDD) + Deep Brain Stimulation [file MDS-36-740-s001.docx]

**Suppl. Table 1- Summary of EKG values for all participants throughout all study visits showing no QTc prolongation in the 150mg nilotinib group**. The baseline QTc range for inclusion into this study was 350-460 ms. An SAE was defined as QTc prolongation ≥ 60 ms from baseline of individual participants AND to a value ≥ 480 ms or QTcF prolongs ≥ 500 ms. ms: millisecond; m: month. NC: non-compliance

| Nilotinib 150mg | Baseline | 1  month | 2 months | 4 months | 6 months | 8 months | 10 months | 12 months | 15 months |
| --- | --- | --- | --- | --- | --- | --- | --- | --- | --- |
| Nil-PD-001 OLE | 430 | 418 | 420 | 442 | 409 | 443 | 441 | 455 | 410 |
| Nil-PD-004 OLE | 422 | 409 | 426 | 420 | 435 | 427 | 435 | 430 | 433 |
| Nil-PD-005 OLE | 416 | 408 | 407 | 415 | 407 | 394 | 406 | 427 | 430 |
| Nil-PD-008 OLE | 429 | 436 | 430 | 433 | 441 | 440 | 434 | 447 | 438 |
| Nil-PD-010 OLE | 430 | 423 | 436 | 439 | 425 | 443 | 426 | 420 | 440 |
| Nil-PD-012 OLE | 413 | 418 | 432 | 414 | 397 | 420 | 450 | 424 | 435 |
| Nil-PD-014 OLE | 420 | 426 | 427 | 425 | 416 | 414 | 418 | 412 | 412 |
| Nil-PD-021 OLE | 439 | 444 | 439 | 457.5 | 436 | 450 | 436 | 473 | NC |
| Nil-PD-022 OLE | 432 | 458 | 462 | 467 | 433 | 438 | 459 | 462 | NC |
| Nil-PD-023 OLE | 404 | 401 | Out (SAE: esophageal carcinoma |  |  |  |  |  |  |
| Nil-PD-027 OLE | 442 | 417 | 420 | 414 | 418 | 401 | 440 | 399 | 442 |
| Nil-PD-028 OLE | 411 | 437 | 448 | 434 | 441 | 432 | 428 | 430 | 446 |
| Nil-PD-029 OLE | 416 | 420 | 419 | 430 | 416 | (SAE)  Cervical injury PI withdrawal |  |  |  |
| Nil-PD-031 OLE | 452 | 437 | 445 | 451 | 437 | 444 | 458 | 458 | 448 |
| Nil-PD-033 OLE | 454 | 441 | 453 | 465 | 468 | 459 | 459 | 460 | 458 |
| Nil-PD-034 OLE | 437 | 410 | 432 | 416 | 424 | 435 | 440 | 449 | 425 |
| Nil-PD-037 OLE | 422 | 420 | 420 | 432 | 431 | 420 | 440 | 435 | 435 |
| Nil-PD-039 OLE | 399 | 407 | 411 | 423 | 417 | 407 | 400 | 390 | 402 |
| Nil-PD-042 OLE | 438 | 403 | 448 | 424 | 430 | 424 | 422 | 464 | 432 |
| Nil-PD-043 OLE | 453 | 457 | 442 | 431 | 462 | 450 | 455 | 490 | tele visit |
| Nil-PD-044 OLE | 428 | 416 | 424 | 425 | 425 | 433 | 426 | 436 | tele visit |
| Nil-PD-045 OLE | 399 | 406 | 403 | 418 | 414 | 429 | 420 | 407 | tele visit |
| Nil-PD-049 OLE | 419 | 418 | 418 | 435 | 422 | 453 | 459 | 457 | 390 |
| Nil-PD-050 OLE | 432 | 416 | 420 | 423 | 418 | 428 | 439 | 425 | N/C |
| Nil-PD-051 OLE | 402 | 394 | 398 | 416 | 410 | 413 | 418 | 392 | 405 |
| Nil-PD-052 OLE | 408 | 427 | 430 | 412 | 406 | 409 | 408 | 423 | 420 |
| Nil-PD-056 OLE | 441 | 426 | 450 | 418 | 424 | 444 | tele visit |  |  |
| Nil-PD-057 OLE | 406 | 399 | 388 | 396 | 405 | 419 | 410 | 405 | tele visit |
| Nil-PD-064 OLE | 432 | 421 | 424 | 404 | 431 | 425 | tele visit |  |  |
| Nil-PD-065 OLE | 411 | 415 | 414 | 426 | 408 | tele visit |  |  |  |
| Nil-PD-069 OLE | 436 | 416 | 431 | 447 | 431 | Self-withdrawal |  |  |  |
| Nil-PD-072 OLE | 428 | 410 | 420 | 429 |  | 427 | 394 | tele visit |  |
| Nil-PD-075 OLE | 426 | 434 | 407 | 441 | 434 | 410 | 425 | tele visit |  |

**Suppl. Table 2- Summary of EKG values for all participants throughout all study visits showing no QTc prolongation in the 300mg nilotinib group.** The baseline QTc range for inclusion into this study was 350-460 ms. An SAE was defined as QTc prolongation ≥ 60 ms from baseline of individual participants AND to a value ≥ 480 ms or QTcF prolongs ≥ 500 ms. ms: millisecond: m: month. NC: non-compliance.

| Nilotinib 300mg | Baseline | 1 month | 2  months | 4  months | 6  months | 8 months | 10 months | 12 months | 15 months |
| --- | --- | --- | --- | --- | --- | --- | --- | --- | --- |
| NIL-PD-002 OLE | 401 | 424 | 430 | 438 | 445 | 453 | self-withdrawal |  |  |
| NIL-PD-003 OLE | 416 | 427 | 422 | 430 | 420 | 425 | 421 | 420 | 426 |
| Nil-PD-006 OLE | 405 | 426 | 429 | 423 | 436 | 404 | 418 | 404 | 402 |
| Nil-PD-007 OLE | 445 | 445 | 412 | 464 | 444 | 442 | 462 | 442 | 445 |
| Nil-PD-009 OLE | 401 | 408 | 386 | 408 | 390 | 411 | 412 | 414 | 418 |
| Nil-PD-013 OLE | 393 | 403 | 407 | 375 | 412 | 449.5 | 405 | 420 | 442 |
| Nil-PD-015 OLE | 416 | 405 | 424 | 435 | 412 | 405 | 423 | 410 | 412 |
| Nil-PD-016 OLE | 414 | 442 | 437 | 460 | 427 | 439 | 469 | 431 | 436 |
| Nil-PD-018 OLE | 407 | 386 | 376 | 401 | 391 | 416 | 410 | 399 | 431 |
| Nil-PD-019 OLE | 470 | 442 | 454 | 456 | 464 | 457 | 453 | 458 | 466 |
| Nil-PD-020 OLE | 450 | 445 | 469 | 452 | 457 | 462 | 466 | 458 | 462 |
| Nil-PD-024 OLE | 455 | 470 | 466 | 465 | 471 | 457 | 466 | 465 | 458 |
| Nil-PD-025 OLE | 420 | 420 | 406 | 424 | 435 | 438 | 442 | 424 | 415 |
| Nil-PD-026 OLE | 410 | Out SAE- NSTEMI |  |  |  |  |  |  |  |
| Nil-PD-032 OLE | 397 | 442 | 442 | 427 | 451 | 426 | 432 | 418 | 432 |
| Nil-PD-035 OLE | 433 | 432 | 431 | 428 | 414 | 414 | 419 | 425 | 427 |
| Nil-PD-036 OLE | 389 | 416 | 385 | 412 | 403 | 402 | 410 | 408 | 413 |
| Nil-PD-038 OLE | 425 | 447 | 425 | 426 | 428 | 429 | 431 | 416 | 413 |
| Nil-PD-040 OLE | 455 | 461 | 444 | 443 | 455 | 444 | 465 | 440 | tele visit |
| Nil-PD-041 OLE | 408 | 398 | 379 | 401 | 363 | 411 | 380 | 395 | 405 |
| Nil-PD-046 OLE | 428 | 441 | 441 | 427 | 452 | 441 | 443 | 436 | 416 |
| Nil-PD-047 OLE | 436 | 420 | 445 | 441 | 429 | 453 | 459 | 457 | tele visit |
| Nil-PD-055 OLE | 423 | 431 | 434 | 441 | 441 | 432 | 427 | tele visit |  |
| Nil-PD-058 OLE | 461 | 447 | 437 | 425 | 442 | 434 | 456 | Out- SAE renal failure |  |
| Nil-PD-059 OLE | 416 | 412 | 429 | 412 | 416 | 421 | 408 | Tele visit |  |
| Nil-PD-060 OLE | 384 | 399 | 409 | 388 | 406 | 417 | 404 | tele visit |  |
| Nil-PD-063 OLE | 404 | 412 | 419 | 407 | 410 | 399 | tele visit | 394 | tele visit |
| Nil-PD-067 OLE | 439 | 460 | 441 | 444 | 455 | 455 | 446 | 457 | tele visit |
| Nil-PD-070 OLE | 455 | 438 | 442 | 429 | 446 | Self-withdrawal |  |  |  |
| Nil-PD-071 OLE | 397 | 406 | 403 | 424 | 445 | NC | 412 | Tele visit |  |

**Mixed: Participants received either placebo, 150mg or 300mg nilotinib in the double-blinded phase (15M) vs drug in OLE (12m)**

**Placebo: Participants received only placebo in the double-blinded phase (15 M) vs drug in OLE (12M)**

**Overlap: Statistics could not be done as some participants were in both groups.**

**Suppl. Table 3-** [Pairwise comparison of changes in clinical endpoints between 150 mg open‐label extension group (mixed to 150 mg) and 300 mg open‐label extension group (mixed to 300 mg) across 2 visits; 15 months-27 months, baseline -15 months, baseline-27 months.](https://wiley.eproofing.in/Proof.aspx?token=e8d9c2388f374ef39ef6a625d160f69a125127179#aq4)The mean changes and 95% Wilcoxon confidence intervals and p-values are summarized.

|  | 27 Months - 15 months | | 15 Months - Baseline | | 27 Months - Baseline | |
| --- | --- | --- | --- | --- | --- | --- |
| **Endpoint** | **mean (95% CI)** | **W p-value** | **mean (95% CI)** | **W p-value** | **mean (95% CI)** | **W p-value** |
| UPDRS I | 0.26 (0, 1) | 0.69 | 0.31 (0, 1) | 0.42 | 0.98 (0, 2) | 0.03 |
| UPDRS II | 2.67 (0, 4) | 0.01 | -0.1 (-2, 1) | 0.64 | 2.62 (0, 5) | 0.06 |
| UPDRS III | 1.29 (-2, 5) | 0.46 | 0.29 (-2, 3) | 0.82 | 2.25 (-2, 6) | 0.29 |
| UPDRS IV | 0.33 (-1, 1) | 0.53 | -0.67 (-2, 0) | 0.1 | -0.13 (-2, 1) | 0.66 |
| UPDRS I-II | 2.93 (0, 5) | 0.02 | 0.21 (-2, 2) | 0.95 | 3.61 (0, 6) | 0.03 |
| UPDRS II-III | 3.96 (-1, 8) | 0.11 | 0.19 (-3, 3) | 0.89 | 4.87 (-1, 10) | 0.12 |
| UPDRS I-II-III | 3.24 (-1, 8) | 0.17 | 0.53 (-3, 4) | 0.74 | 4.96 (-1, 12) | 0.12 |
| PDQ39 | 9.91 (0, 16) | 0.04 | -0.16 (-8, 10) | 0.76 | 9.8 (0, 21) | 0.06 |
| MOCA | -0.87 (-2, 0) | 0.09 | 0.24 (-1, 1) | 0.82 | -0.25 (-1, 1) | 0.72 |

**Suppl. Table 4-**  Mean changes in clinical endpoints **within** each group across two visits, 15 months and 27 months, between baseline and 15 months, and baseline and 27 months. Comparison were performed using Wilcoxon rank-sum test and their 95% confidence intervals. Group A represents placebo to 150mg (n=9), B represents placebo to 300mg (n=13), C represents 150mg to 150mg (n=11), D represents 300mg to 300mg (n=6), E represents 150mg to 300mg (n=9), F represents 300mg to 150mg (n=11).

|  |  | 27 Months-15 Months |  | 15 Months- Baseline |  | 27 Months - Baseline |  |
| --- | --- | --- | --- | --- | --- | --- | --- |
| **Endpoint** | **Group** | mean (95% CI) | W p-value | mean (95% CI) | W p-value | mean (95% CI) | W p-value |
| UPDRS I | A | 1 (3, 3) | 0.17 | -0.111 (-2.5, 2.5) | 0.93 | 1.143 (0.5, 3) | 0.13 |
|  | B | -0.091 (-1, 1) | 0.82 | 0.308 (-1, 2) | 0.41 | 0.182 (-1, 1) | 0.80 |
|  | C | 0.4 (-1, 2) | 0.41 | 0.636 (1, 2) | 0.18 | 1.3 (0.5, 3) | 0.04 |
|  | D | 0.75 (-1, 3) | 0.59 | -0.5 (-1.5, 0.5) | 0.49 | 0 (0, 0) | 1.00 |
|  | E | 0 (0, 0) | 1.00 | 0.333 (-1, 1.5) | 0.37 | 0 (-1, 1) | 1.00 |
|  | F | -0.25 (-1.5, 1) | 0.67 | 0.727 (-1, 2.5) | 0.22 | 0.75 (-1, 2.5) | 0.20 |
| UPDRS II | A | 3.857 (0, 7.5) | 0.04 | 2.667 (-0.5, 6.5) | 0.11 | 6.429 (3, 12) | 0.06 |
|  | B | -1.455 (-4, 2) | 0.30 | 3.154 (2.5, 5.5) | 0.01 | 2.091 (-1.5, 6) | 0.14 |
|  | C | 1.5 (-1, 3.5) | 0.14 | 1.545 (0, 4) | 0.06 | 3.3 (1, 6) | 0.01 |
|  | D | NA | 0.35 | 2.833 (1, 6) | 0.14 | 2 (-1, 6) | 0.42 |
|  | E | 0.5 (-2, 4) | 0.79 | 1 (-2, 3) | 0.37 | 2.167 (-3, 6) | 0.18 |
|  | F | NA | 0.05 | 2.727 (1, 6) | 0.02 | 5 (3.5, 8.5) | 0.02 |
| UPDRS III | A | 3.714 (-1, 9) | 0.14 | -1.333 (-4.5, 2) | 0.41 | 2.143 (-4, 9.5) | 0.67 |
|  | B | -1.545 (-6, 3) | 0.28 | -1.538 (-5.5, 2) | 0.21 | -3.364 (-8, 1) | 0.14 |
|  | C | 2.3 (-3, 8) | 0.23 | -2.545 (-7.5, 3) | 0.11 | -0.2 (-5.5, 5) | 1.00 |
|  | D | 1.75 (3.5, 3.5) | 0.37 | -1.833 (-6, 1) | 0.50 | -1.25 (-3, 2) | 0.58 |
|  | E | -0.333 (-4.5, 5) | 1.00 | -2.778 (-7, 1.5) | 0.15 | -3.667 (-9, 1) | 0.25 |
|  | F | -3.875 (-9, 0) | 0.11 | -1.182 (-4, 2) | 0.35 | -4.125 (-10.5, 1) | 0.08 |
| UPDRS IV | A | -0.571 (-2, -0.5) | 0.27 | 0 (-1.5, 2) | 0.58 | -0.571 (-3, 1.5) | 0.60 |
|  | B | -0.727 (-3.5, 1.5) | 0.23 | 0.769 (-1, 3) | 0.35 | 0.182 (-1, 2) | 0.72 |
|  | C | 0.4 (-1.5, 2.5) | 0.61 | 0.091 (-1.5, 2) | 0.94 | 0.7 (-1.5, 3) | 0.47 |
|  | D | -0.25 (-1, -1) | 0.77 | 0.167 (-2.5, 2) | 0.40 | -0.75 (-6, 2) | 1.00 |
|  | E | 0.5 (0, 1.5) | 0.35 | 0.667 (-0.5, 2) | 0.25 | 1.167 (0, 2) | 0.11 |
|  | F | 0.125 (-1, 1.5) | 0.75 | -0.273 (-3.5, 2.5) | 0.92 | 0.125 (-4, 3) | 0.49 |
| UPDRS I-II | A | 4.857 (2, 8) | 0.02 | 2.556 (-2, 8.5) | 0.23 | 7.571 (4, 14) | 0.06 |
|  | B | -1.545 (-5.5, 2) | 0.38 | 3.462 (2, 5.5) | 0.00 | 2.273 (-2, 7) | 0.19 |
|  | C | 1.9 (-1, 5) | 0.08 | 2.182 (0, 4) | 0.04 | 4.6 (2, 8) | 0.01 |
|  | D | 1.75 (-1, 5) | 0.42 | 2.333 (-2, 7) | 0.20 | 2 (-3, 6) | 0.42 |
|  | E | 0.5 (-2, 5) | 1.00 | 1.333 (-1, 4) | 0.28 | 2.167 (-1.5, 6) | 0.25 |
|  | F | 1.5 (0.5, 4) | 0.07 | 3.455 (1, 8) | 0.03 | 5.75 (3, 10.5) | 0.02 |
| UPDRS II-III | A | 7.571 (0, 15.5) | 0.04 | 1.333 (-4, 6.5) | 0.68 | 8.571 (-3.5, 21) | 0.16 |
|  | B | -3 (-7, 1) | 0.11 | 1.615 (-2, 5) | 0.48 | -1.273 (-6.5, 4) | 0.51 |
|  | C | 3.8 (-2, 11) | 0.21 | -1 (-5, 3) | 0.53 | 3.1 (-5, 10) | 0.41 |
|  | D | 2.75 (5.5, 5.5) | 0.37 | 1 (-6, 6) | 0.75 | 0.75 (-7, 8) | 0.79 |
|  | E | 0.167 (-8, 5) | 0.86 | -1.778 (-6.5, 2.5) | 0.40 | -1.5 (-9.5, 6) | 1.00 |
|  | F | -2.125 (-8, 2) | 0.45 | 1.545 (-2.5, 5) | 0.48 | 0.875 (-6, 9) | 0.83 |
| UPDRS I-II-III | A | 8.571 (4.5, 15.5) | 0.04 | 1.222 (-5, 7.5) | 0.68 | 9.714 (-7, 23.5) | 0.13 |
|  | B | -2.909 (-8.5, 1.5) | 0.11 | 1.923 (-1.5, 6) | 0.25 | -0.909 (-6, 3.5) | 0.62 |
|  | C | 4.2 (-2, 12.5) | 0.17 | -0.273 (-6, 5.5) | 0.96 | 4.5 (-3.5, 12) | 0.18 |
|  | D | 3.75 (-1, 9) | 0.27 | 0.333 (-7, 6) | 0.92 | 0.75 (-3.5, 5) | 0.85 |
|  | E | 0.167 (-9, 5) | 0.86 | -1.444 (-7.5, 4.5) | 0.62 | -1.5 (-10.5, 7) | 1.00 |
|  | F | -5 (-14, 3) | 0.18 | 2.182 (-2.5, 7) | 0.33 | -1 (-13.5, 10.5) | 1.00 |
| PDQ-39 | A | 13 (2.5, 30) | 0.09 | 5.333 (-4, 14) | 0.21 | 16.286 (-7, 34) | 0.11 |
|  | B | -7.909 (-27.5, 5) | 0.29 | 11.462 (1, 21) | 0.04 | 6.909 (-7.5, 19.5) | 0.20 |
|  | C | 4.2 (-4, 12.5) | 0.26 | 3.182 (-6, 13) | 0.45 | 8.3 (-5, 21.5) | 0.19 |
|  | D | NA | 1.00 | 7.667 (-12, 51) | 0.84 | -5.75 (-12, 3) | 0.25 |
|  | E | -3.167 (-14, 7) | 0.67 | 0.111 (-15, 15.5) | 1.00 | -3.5 (-23, 11) | 0.56 |
|  | F | -1.875 (-13, 11) | 0.67 | 11.727 (6, 18.5) | 0.00 | 10.75 (-3, 22.5) | 0.08 |
| MoCA | A | -1.286 (-3, -1.5) | 0.10 | 0 (-2, 2) | 1.00 | -1.286 (-4, 1) | 0.28 |
|  | B | -0.182 (-2, 2) | 0.83 | -0.308 (-1.5, 1) | 0.31 | -0.455 (-2.5, 1) | 0.43 |
|  | C | -1.1 (-3, -0.5) | 0.07 | -0.091 (-2, 1.5) | 0.67 | -1 (-3, 0.5) | 0.17 |
|  | D | NA | 0.35 | -0.833 (-3, 1) | 0.58 | -1.75 (-6, 1) | 0.42 |
|  | E | -0.167 (-1, 1) | 0.85 | 0.444 (-2, 5) | 0.78 | NA | 0.15 |
|  | F | -1.25 (-4, -1) | 0.10 | 0.273 (-1, 2) | 0.58 | -0.625 (-3, 1) | 0.27 |

**Suppl. Table 5-** [Pairwise comparison of changes in clinical endpoints between groups A (placebo to 150 mg) and B (placebo to 300 mg), groups A and C (150 mg to 150 mg), and groups B and D (300 mg to 300 mg), across 2 visits; 15 months -27 months, baseline -15 months, baseline -27 months.”](https://wiley.eproofing.in/Proof.aspx?token=e8d9c2388f374ef39ef6a625d160f69a125127179#aq5)The mean changes and 95% Wilcoxon confidence intervals and p-values are summarized.

|  |  | 27 Months - 15 months | | 15 Months - Baseline | | 27 Months - Baseline | |
| --- | --- | --- | --- | --- | --- | --- | --- |
| **Endpoint** | **Groups** | **mean (95% CI)** | **W p-value** | **mean (95% CI)** | **W p-value** | **mean (95% CI)** | **W p-value** |
| UPDRS I | A vs B | 1.09 (0, 3) | 0.16 | -0.42 (-2, 1) | 0.51 | 0.96 (-1, 3) | 0.25 |
|  | A vs C | 0.6 (0, 2) | 0.34 | -0.75 (-2, 1) | 0.31 | -0.16 (-2, 2) | 0.88 |
|  | B vs D | -0.84 (-3, 1) | 0.45 | 0.81 (0, 2) | 0.13 | 0.18 (-2, 3) | 0.89 |
| UPDRS II | A vs B | 5.31 (1, 9) | 0.02 | -0.49 (-4, 2) | 0.61 | 4.34 (-2, 11) | 0.13 |
|  | A vs C | 2.36 (-1, 5) | 0.16 | 1.12 (-2, 4) | 0.52 | 3.13 (-3, 9) | 0.35 |
|  | B vs D | -2.45 (-7, 2) | 0.26 | 0.32 (-3, 4) | 0.76 | 0.09 (-5, 6) | 1.00 |
| UPDRS III | A vs B | 5.26 (0, 11) | 0.06 | 0.21 (-4, 5) | 0.97 | 5.51 (-2, 11) | 0.26 |
|  | A vs C | 1.41 (-5, 8) | 0.69 | 1.21 (-4, 6) | 0.68 | 2.34 (-6, 9) | 0.66 |
|  | B vs D | -3.3 (-9, 2) | 0.15 | 0.29 (-5, 5) | 0.96 | -2.11 (-10, 5) | 0.55 |
| UPDRS IV | A vs B | 0.16 (-1, 2) | 0.78 | -0.77 (-2, 1) | 0.27 | -0.75 (-3, 2) | 0.41 |
|  | A vs C | -0.97 (-3, 1) | 0.31 | -0.09 (-2, 1) | 0.64 | -1.27 (-4, 1) | 0.37 |
|  | B vs D | -0.48 (-3, 1) | 0.55 | 0.6 (-2, 5) | 0.53 | 0.93 (-3, 6) | 0.89 |
| UPDRS I-II | A vs B | 6.4 (2, 11) | 0.01 | -0.91 (-5, 2) | 0.42 | 5.3 (-1, 12) | 0.12 |
|  | A vs C | 2.96 (0, 7) | 0.08 | 0.37 (-4, 4) | 0.79 | 2.97 (-4, 10) | 0.43 |
|  | B vs D | -3.3 (-9, 2) | 0.26 | 1.13 (-2, 5) | 0.43 | 0.27 (-6, 8) | 0.90 |
| UPDRS II-III | A vs B | 10.57 (3, 19) | 0.01 | -0.28 (-7, 6) | 0.87 | 9.84 (-3, 22) | 0.11 |
|  | A vs C | 3.77 (-5, 14) | 0.30 | 2.33 (-4, 9) | 0.38 | 5.47 (-8, 17) | 0.35 |
|  | B vs D | -5.75 (-12, 1) | 0.07 | 0.62 (-7, 8) | 1.00 | -2.02 (-11, 7) | 0.60 |
| UPDRS I-II-III | A vs B | 11.48 (4, 19) | 0.01 | -0.7 (-8, 6) | 1.00 | 10.62 (-2, 24) | 0.11 |
|  | A vs C | 4.37 (-4, 14) | 0.24 | 1.49 (-5, 9) | 0.65 | 5.21 (-9, 18) | 0.41 |
|  | B vs D | -6.66 (-15, 1) | 0.08 | 1.59 (-6, 9) | 0.76 | -1.66 (-11, 8) | 0.74 |
| PDQ39 | A vs B | 20.91 (3, 37) | 0.03 | -6.13 (-18, 11) | 0.33 | 9.38 (-13, 31) | 0.30 |
|  | A vs C | 8.8 (-7, 25) | 0.22 | 2.15 (-10, 14) | 0.70 | 7.99 (-16, 32) | 0.35 |
|  | B vs D | -7.16 (-31, 10) | 0.64 | 3.79 (-13, 27) | 0.46 | 12.66 (-8, 34) | 0.17 |
| MOCA | A vs B | -1.1 (-2, 0) | 0.20 | 0.31 (-1, 2) | 0.65 | -0.83 (-3, 1) | 0.64 |
|  | A vs C | -0.19 (-2, 2) | 0.80 | 0.09 (-1, 2) | 0.75 | -0.29 (-3, 2) | 0.96 |
|  | B vs D | 0.82 (-1, 2) | 0.37 | 0.53 (-1, 3) | 0.96 | 1.3 (-2, 6) | 0.55 |

| **Levodopa Equivalent Daily Dose (LEDD) in mg** | **Baseline Mean±SD** | **15 Months**  **Mean±SD** | **27 Months Mean±SD** | | **Difference**  **Baseline-27 months (%)** | **No. of Participants on DBS (%)** | **No. of Participants on AChEI (%)** |
| --- | --- | --- | --- | --- | --- | --- | --- |
| **A- placebo to 150mg (n=7)** | **778±308** | **877±320** | **950±291** | **172 mg (22%)** | | **2 (28%)** | **4 (12%)** |
| **B- placebo to 300mg (n=11)** | **668±284** | **716±263** | **795±276** | **127 mg (19%)** | | **2 (18%)** | **1 (3%)** |
| **C- Mixed to 150mg (n-25)** | **749±261** | **878±304** | **945±341** | **196mg (26%)** | | **6 (24%)** | **8 (24%)** |
| **D- Mixed to 300mg (n=21)** | **728±381** | **798±388** | **941±411** | **213 mg (30%)** | | **5 (24%)** | **2 (6%)** |

**Suppl. Table 6: Levodopa Equivalent Daily Dose (LEDD) + Deep Brain Stimulation (DBS) + Acetylcholinesterase inhibitors (AChEI) in each group. (**A) placebo to 150mg, (B) placebo to 300mg, (C) mixed to 150mg, (D) mixed to 300mg nilotinib. Mixed: Participants received either placebo, 150mg or 300mg nilotinib in the double-blinded phase (15M) vs drug in OLE (12m). Placebo: Participants received only placebo in the double-blinded phase (15 M) vs drug in OLE (12M).
